# Conventional and novel [^18^F]FDG PET/CT features as predictors of CAR-T cell therapy outcome in large B-cell lymphoma

**DOI:** 10.1186/s13045-024-01540-x

**Published:** 2024-04-23

**Authors:** Doris Leithner, Jessica R. Flynn, Sean M. Devlin, Audrey Mauguen, Teng Fei, Shang Zeng, Junting Zheng, Brandon S. Imber, Harper Hubbeling, Marius E. Mayerhoefer, Akshay Bedmutha, Efrat Luttwak, Magdalena Corona, Parastoo B. Dahi, Alejandro Luna de Abia, Ivan Landego, Richard J. Lin, M. Lia Palomba, Michael Scordo, Jae H. Park, Ana Alarcon Tomas, Gilles Salles, Daniel Lafontaine, Laure Michaud, Gunjan L. Shah, Miguel-Angel Perales, Roni Shouval, Heiko Schöder

**Affiliations:** 1 Department of Radiology, Memorial Sloan Kettering Cancer Center, New York, USA; 2 Department of Radiology, NYU Grossman School of Medicine, New York, USA; 3 Department of Epidemiology and Biostatistics, Memorial Sloan Kettering Cancer Center, New York, NY USA; 4 Department of Radiation Oncology, Memorial Sloan Kettering Cancer Center, New York, NY USA; 5 Department of Biomedical Imaging and Image-guided Therapy, Medical University of Vienna, Vienna, Austria; 6 Department of Medicine, Adult Bone Marrow Transplantation Service, Memorial Sloan Kettering Cancer Center, 530 E74th Street, NY 10021 New York, USA; 7 Department of Medicine, Lymphoma Service, Memorial Sloan Kettering Cancer Center, New York, NY USA; 8 Department of Medicine, Weill Cornell Medical College, New York, NY USA; 9 Hematology and Hemotherapy Service, Hospital Universitario 12 de Octubre, Madrid, Spain; 10 Bone Marrow Transplantation Unit, Hematology Service, Hospital Universitario Ramón y Cajal, Madrid, Spain; 11 Department of Medicine, Leukemia Service, Memorial Sloan Kettering Cancer Center, New York, NY USA; 12 Department of Hematology, Hospital Universitario Puerta de Hierro, Madrid, Spain; 13 Department of Medical Physics, Memorial Sloan Kettering Cancer Center, New York, USA; 14 Department of Nuclear Medicine and Molecular ImagingLausanne University Hospital (CHUV), Lausanne, Switzerland

**Keywords:** Lymphoma, Positron emission tomography, Biomarker, Immunotherapy, CAR-T, Radiomics

## Abstract

Relapse and toxicity limit the effectiveness of chimeric antigen receptor T-cell (CAR-T) therapy for large B-cell lymphoma (LBCL), yet biomarkers that predict outcomes and toxicity are lacking. We examined radiomic features extracted from pre-CAR-T ^18^F-fluorodeoxyglucose positron emission tomography/computed tomography ([^18^F]FDG PET/CT) scans (*n* = 341) of 180 patients (121 male; median age, 66 years). Three conventional (maximum standardized uptake value [SUVmax], metabolic tumor volume [MTV], total lesion glycolysis [TLG]) and 116 novel radiomic features were assessed, along with inflammatory markers, toxicities, and outcomes. At both pre-apheresis and pre-infusion time points, conventional PET features of disease correlated with elevated inflammatory markers. At pre-infusion, MTV was associated with grade ≥ 2 cytokine release syndrome (odds ratio [OR] for 100 mL increase: 1.08 [95% confidence interval (CI), 1.01–1.20], *P* = 0.031), and SUVmax was associated with failure to achieve complete response (CR) (OR 1.72 [95% CI, 1.24–2.43], *P* < 0.001). Higher pre-apheresis and pre-infusion MTV values were associated with shorter progression-free survival (PFS) (HR for 10-unit increase: 1.11 [95% CI, 1.05–1.17], *P* < 0.001; 1.04 [95% CI, 1.02–1.07], *P* < 0.001) and shorter overall survival (HR for 100-unit increase: 1.14 [95% CI, 1.07–1.21], *P* < 0.001; 1.04 [95% CI, 1.02–1.06], *P* < 0.001). A combined MTV and LDH measure stratified patients into high and low PFS risk groups. Multiple pre-infusion novel radiomic features were associated with CR. These quantitative conventional [^18^F]FDG PET/CT features obtained before CAR-T cell infusion, which were correlated with inflammation markers, may provide prognostic biomarkers for CAR-T therapy efficacy and toxicity. The use of conventional and novel radiomic features may thus help identify high-risk patients for earlier interventions.

**Supplementary Information:**

The online version contains supplementary material available at 10.1186/s13045-024-01540-x.

Among the emerging treatments for patients with large-B cell lymphoma (LBCL), CD19-directed chimeric antigen receptor T cell (CAR-T) therapy demonstrates potential for sustained disease remission [1, 2]. However, 60% of patients treated with CAR-T cells experience disease relapse or progression within 6 months [3, 4], and severe therapy-associated toxicities, such as cytokine release syndrome (CRS) and neurotoxicity, are common [5, 6]. Therefore, biomarkers that predict the risk of treatment failure, and could trigger early on-treatment interventions, are urgently needed. ^18^F-fluorodeoxyglucose positron emission tomography/computed tomography ([^18^F]FDG PET/CT) is the standard-of-care for staging and response assessment of LBCL [7], but its role in guiding CAR-T therapy is not fully explored. Prior to CAR-T therapy, patients may undergo PET/CT before leukapheresis, to assess treatment eligibility, disease extent, and the need for bridging therapy; and again before lymphodepletion and CAR-T cell infusion, as baseline for response assessment. Previous research suggested that these PET scans [8], as well as certain laboratory parameters [6, 9], may be prognostic for therapy outcomes and treatment-associated toxicities. In our study–the largest to-date on this topic– we investigated (1) whether conventional and novel radiomic [^18^F]FDG PET/CT features could predict treatment response, survival, and treatment-related toxicities in patients with LBCL receiving CAR-T therapy; and (2) for the first time, whether laboratory markers of inflammation are correlated with PET features.

## Findings

**Fig. 1:**
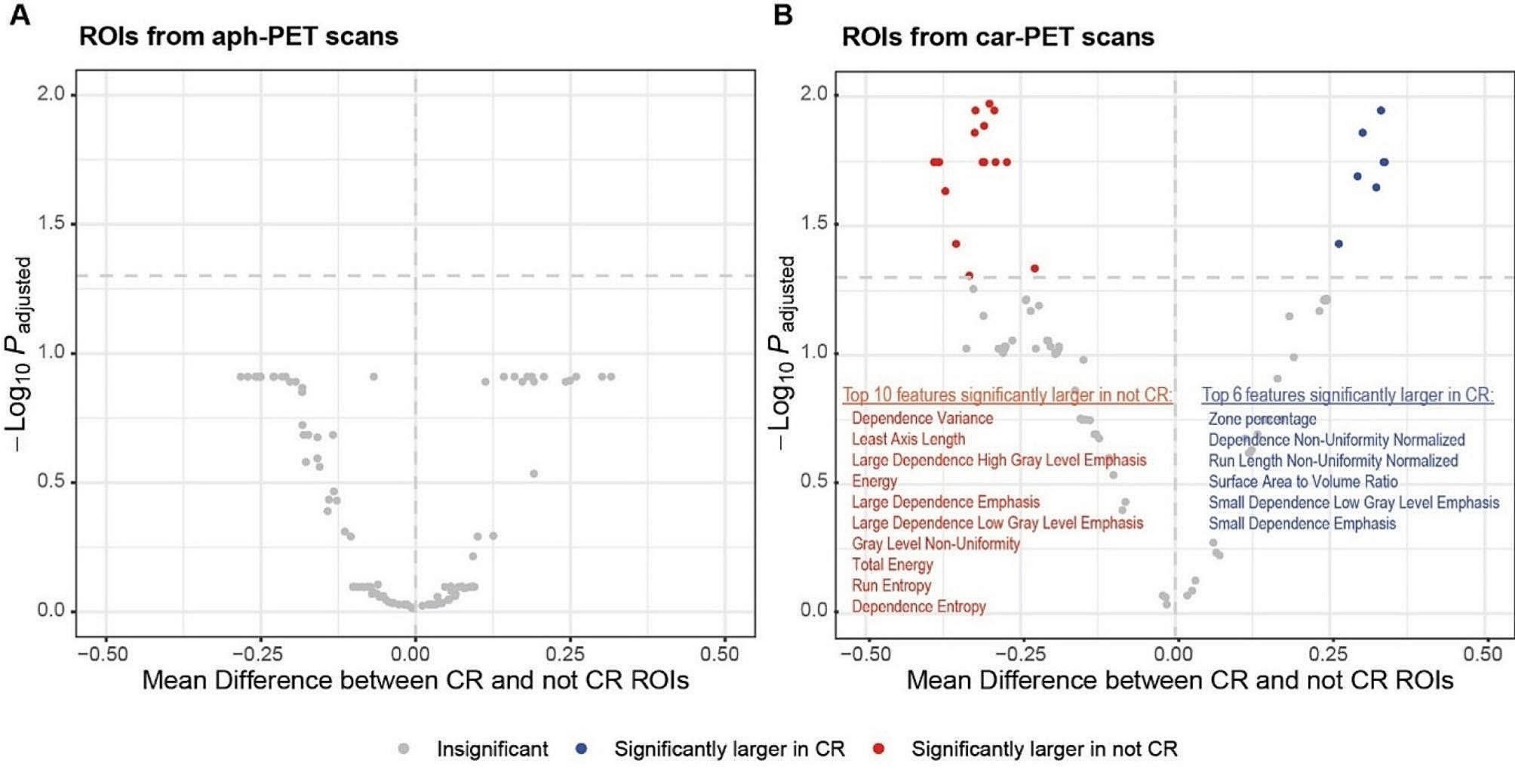
**Prognostic value of PET radiomic features. **Volcano plots showing PET radiomic features from (**A**) aph-PET and (**B**) car-PET scans. Radiomic features extracted from aph-PET scans did not differ significantly between patients achieving complete remission (CR) or not. In contrast, several radiomic features extracted from lymphoma manifestations on car-PET scans, such as Energy and Zone Percentage, differed significantly (*P* < 0.05) between the two outcome groups (CR vs. non-CR). Abbreviations: Aph-PET = pre-leukapheresis PET scan; Car-PET = pre-CAR-T cell infusion PET scan; CR = complete response; ROI = region of interest

**Fig. 2:**
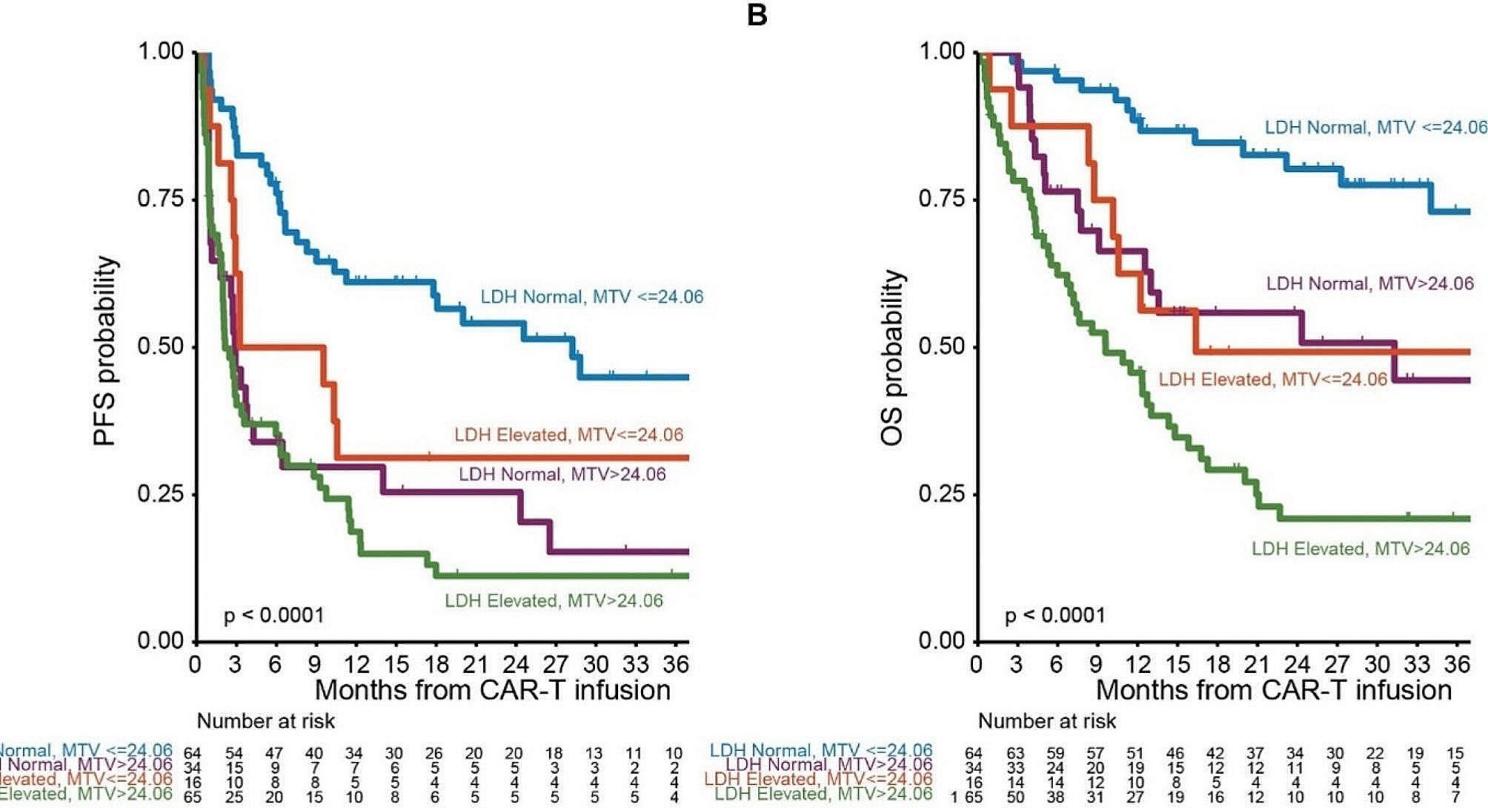
**Combined prognostic value of MTV and LDH**. Kaplan-Meier curves show that the combination of car-PET MTV (based on a cutoff of 24.06) and LDH separate high and low (**A**) progression-free survival (PFS) and (**B**) overall survival (OS) risk groups. Abbreviations: LDH = lactate dehydrogenase; MTV = metabolic tumor volume

We retrospectively included 180 patients with LBCL who had undergone [^18^F]FDG PET/CT at pre-apheresis (aph-PET) and/or pre-infusion (car-PET) time points before autologous CD19-directed CAR-T therapy at Memorial Sloan Kettering Cancer Center (axi-cel, *n* = 93; tisa-cel, *n* = 52; and liso-cel, *n* = 35 patients; Additional Files 1 and 2). In total, 341 PET/CT scans (161 aph-PET and 180 car-PET scans) performed on different PET scanners were analyzed; some car-PETs were performed before apheresis and thus considered for two time points. Maximum standardized uptake value (SUVmax), metabolic tumor volume (MTV), total lesion glycolysis (TLG), and 116 radiomic features capturing metabolic heterogeneity and lesion shape, were calculated for each PET/CT (Additional File 1). Markers of tumor burden and inflammation (LDH, CRP, IL-6, IL-10, TNF-α, ferritin, fibrinogen, D-dimer) were correlated with aph-PET and car-PET features (Additional Files 2 and 3). Following multivariable adjustment, car-PET MTV showed a significant association with CRS (OR 1.08 for 100-unit increase [95% CI, 1.01–1.20], *P* = 0.031) (Additional File 4). Failure to achieve complete remission (CR) after CAR-T therapy was associated with higher car-PET SUVmax (OR 1.72 for 10-unit increase [95% CI, 1.24–2.43], *P* < 0.001). Of 116 car-PET radiomic features, 47 differed significantly between patients with, and those without, day 100 best response CR (adjusted *P* < 0.05) (Fig. 1). Higher aph-PET MTV (HR 1.11 for 10-unit increase [95% CI, 1.05–1.17], *P* < 0.001) and car-PET MTV (HR 1.04 for 10-unit increase [95% CI, 1.02–1.07], *P* < 0.001) were associated with shorter PFS. Similarly, higher aph-PET MTV (HR 1.14 for 100-unit increase [95% CI, 1.07–1.21], *P* < 0.001) and car-PET MTV (HR 1.04 for 100-unit increase [95% CI, 1.02–1.06], *P* < 0.001) were associated with shorter OS. The combination of MTV (calculated cutoff, 24 mL) and LDH, both significant outcome predictors on multivariable analysis, enabled separation of high and low PFS and OS risk groups, and two intermediate risk groups (Fig. 2).

## Discussion

CAR-T cell response, survival, and toxicities are clinically relevant endpoints for which predictive biomarkers are currently lacking. Our data suggest that higher car-PET SUVmax may be associated with higher likelihood of non-CR to CAR-T cells. This finding, which has not been reported before, may be explained by the prior observation that SUV on [^18^F]FDG-PET is linked to lymphoma aggressiveness. Moreover, we found significant differences in several car-PET radiomic features between patients achieving, and those not achieving, CR. These features quantitatively assess lesion heterogeneity and shape, and have previously shown correlations with tumor aggressiveness and clinical outcomes [10]. Our identification of MTV as a key parameter associated with poor PFS and OS in patients treated with CAR-T cells confirms the findings of prior smaller studies [8, 11]. With regard to toxicities–a major limiting factor for CAR-T therapy– we found that car-PET MTV may predict the development of CRS. We also observed associations between car-PET and aph-PET features and multiple inflammation markers that are linked to an immunosuppressive tumor microenvironment, and thus, probably also to toxicities and lower response rates to CAR-T therapy [9].

The present study has some limitations. First, radiomics is still an exploratory analytic technique whose results are influenced by multiple factors, such as acquisition parameters [12]. While radiomic feature extraction is relatively fast (approximately 5 min per PET/CT) and, per se, reproducible, interrater differences in terms of lesion delineation/segmentation are known to affect feature values [10]. Since we did not further evaluate our model performance by cross-validation or in a held-out cohort, the results of our radiomic analyses must be considered as preliminary and require external validation. Second, our risk classification model combines MTV and LDH, both of which (directly or indirectly) reflect tumor volume; our model illustrates how MTV could further improve risk definition from LDH alone, and therefore the incremental utility of PET parameters. Third, data on extra-nodal involvement, which is a known risk factor in CAR-T cell therapy, was not available in this study and might be of interest in further analyses. Fourth, some car-PETs were considered for two time points, rendering our cohort more heterogeneous due to possible bridging therapies. In conclusion, for patients with LBCL undergoing CAR-T therapy, quantitative [^18^F]FDG PET/CT features assessed immediately before CAR-T cell infusion are associated with clinical outcomes, treatment response, toxicity, and markers of inflammation. [^18^F]FDG PET/CT features could therefore guide additional interventions in high-risk populations to increase the efficacy and safety of CAR-T therapy.

### Electronic supplementary material

Below is the link to the electronic supplementary material.

Supplementary Material 1

Supplementary Material 2

Supplementary Material 3

Supplementary Material 4

Supplementary Material 5

Supplementary Material 6

Supplementary Material 7

## Data Availability

The datasets used and analysed during the current study are available from the corresponding author on reasonable request.

## Abbreviations

Aph-PET: Last PET before leukapheresis
Car-PET: Last PET before lymphodepletion and CAR-T cell infusion
CAR-T: CD19-directed chimeric antigen receptor T cell
CR: Complete response
CRP: C-reactive protein
CRS: Cytokine release syndrome
IL-6: Interleukin 6
IL-10: Interleukin 10
LBCL: Large-B cell lymphoma
LDH: Lactate dehydrogenase
MTV: Metabolic tumor volume
OS: Overall survival
PET/CT: Positron emission tomography/computed tomography
PFS: Progression-free survival
SUV: Standardized uptake value
TLG: Total lesion glycolysis
TNF-α: Tumor necrosis factor alpha
[^18^F]FDG: ^18^F-fluorodeoxyglucose
