# Supplementary material for: Conventional and novel [18F]FDG PET/CT features as predictors of CAR-T cell therapy outcome in large B-cell lymphoma
Source: J Hematol Oncol. 2024 Apr 23;17:21. doi: 10.1186/s13045-024-01540-x (PMC11035117; doi:10.1186/s13045-024-01540-x)
Supplement: Supplementary file 1 — Supplementary Material 1 [file 13045_2024_1540_MOESM1_ESM.docx]

**ADDITIONAL FILE 1**

**Supplementary Materials and Methods**

**Patient cohort and design**

Adult patients (age ≥18 years) with de novo or transformed LBCL who had undergone [^18^F]FDG PET/CT prior to autologous CD19-directed CAR-T therapy (axicabtagene ciloleucel [axi-cel], tisagenlecleucel [tisa-cel], and lisocabtagene maraleucel [liso-cel]) at Memorial Sloan Kettering Cancer Center (MSK) between 04/2016 and 10/2022 and had undergone [^18^F]FDG PET/CT at pre-apheresis and/or pre-infusion time points were included. CAR-T products were administered as standard of care, except for liso-cel infusions, some of which were performed as part of a clinical trial (NCT02631044) [1]. Patients with central nervous system involvement at the time of apheresis or with primary intravascular lymphoma were excluded. Patient data were manually recorded and entered into a REDCap database [2]. Pre-CAR-T infusion laboratory measurements, including C-reactive protein (CRP), interleukin 6 (IL-6), IL-10, tumor necrosis factor alpha (TNF-α), lactate dehydrogenase (LDH), ferritin, fibrinogen, and D-dimer, were retrieved from electronic medical records; time points for PET imaging and cytokine measurements were matched. This study was approved by the local Institutional Review Board; all patients signed informed consent for treatment and data collection.  **Treatment response and outcome**

Response to CAR-T therapy was defined according to the Lugano classification [3]. All patients were followed up with [^18^F]FDG PET/CT after CAR-T infusion; PET-based response was regularly assessable and all examinations appropriate for response assessment with Lugano criteria. CRS and immune effector cell-associated neurotoxicity syndrome (ICANS) were graded according to American Society of Transplant and Cellular Therapy grading criteria [4]. Overall survival (OS) and progression-free survival (PFS) were measured from the time of CAR-T cell infusion; corresponding events were death for OS and death, relapse, or disease progression for PFS. Patients without events were censored at their date of last follow-up.

**PET imaging and feature extraction**

PET/CT at MSK was performed on Discovery 690 and Discovery 710 scanners (GE Healthcare, Waukesha, WI) after intravenous injection of 444 MBq ± 10% of [^18^F]FDG. Low-dose CT (120-140kV; 80mA) and PET emission scans (2-3 min/bed position) of the torso (skull base to upper thigh) were obtained following an uptake time of about 1 hour. The low-dose, non-contrast-enhanced CT scan was used for attenuation correction and anatomical correlation. A heavy z-axis filter and Gaussian transaxial filter with 6.4 mm cutoff was used. Blood glucose levels were <180 mg/dL prior to PET. All scans passed visual quality control.

Scans at 2 time points were considered:

(1) **aph-PET**: last PET before leukapheresis.

(2) **car-PET**: last PET before lymphodepletion and CAR-T cell infusion, which includes post-apheresis and post-bridging scans, as well as pre-apheresis for patients without additional imaging afterwards.

The median for the time between aph-PET and apheresis was 21 days (interquartile range [IQR]: 12-32). The median for the time between car-PET and infusion was 15 days (IQR: 10-40). Using the Beth-Israel PET/CT viewer plugin for FIJI [5], maximum standardized uptake value (SUVmax) and metabolic tumor volume (MTV) were calculated semi-automatically by a board-certified radiologist using fixed lower and upper thresholds for SUV of 4-200, as recommended in prior studies [6, 7]; the reader had access to current, prior, and follow-up imaging data and reports. Total lesion glycolysis (TLG), i.e., the product of MTV and SUVmean, were calculated. The maximum lesion diameter was measured on CT transaxial or coronal planes, as previously recommended [8], in the Hermes Viewer software (Hermes Medical Solutions, Stockholm, Sweden). The presence of nodes/nodal conglomerates of >4 cm was recorded and subdivided into 2 categories of bulky disease: 6-10 cm and >10 cm [3]. Using the International Biomarker Standardization Initiative compliant PyRadiomics plugin for FIJI [9], 3-dimensional radiomic features were extracted on a per-lesion basis from all scans with measurable MTV. Before radiomic feature extraction, intensity discretization using a fixed bin width of 0.5 and spatial resampling to 1.5 x 1.5 x 1.5 mm^3^ voxels using B-spline interpolation were applied, as described previously [10]. Radiomic features were derived from the gray-level histogram, co-occurrence matrix, run-length matrix, size-zone matrix, neighboring gray-tone difference matrix, and lesion shape. Nineteen gray-level histogram, 26 shape-based, 24 co-occurrence matrix, 16 run-length matrix, 16 size-zone matrix, 5 neighboring gray-tone difference matrix and 10 conventional and segmentation-related features from segmentation files, were calculated (n=116 radiomic features; equations used for feature calculations are available at https://pyradiomics.readthedocs.io/en/latest/features.html).

**Statistical analysis**

Continuous variables were described using median and range, and categorical data presented as frequencies and percentages. Associations between PET imaging features and markers of inflammation were evaluated using Spearman rank correlations. Associations with outcomes of OS and PFS were analyzed using Cox proportional hazard models, and associations with grade 2-4 CRS, grade 2-4 ICANS, and complete response (CR) were analyzed using logistic regression models. Multivariable analyses for OS and PFS were stratified by bridging status. Parameters with significant *P*-values on univariable analysis were included in multivariable analyses, with the exception of highly correlated features, such as MTV, TLG, and bulk, where only MTV was included. Other relevant variables, such as CAR-T costimulatory domain and patient age, were included in multivariable analyses irrespective of *P*-value due to their clinical importance. Median follow-up was calculated by reversed Kaplan-Meier estimator. An optimal MTV cutpoint was found using maximally selected rank statistics [11]. Multivariable generalized estimating equation (GEE) models were applied to evaluate differential expression of radiomic features between regions of interest (ROIs) from individuals with day 100 CR and those with non-CR, adjusted for age, co-stimulatory domains, pre-lymphodepletion LDH, and non-Hodgkin lymphoma (NHL) subtype. Here, each radiomic feature was first scaled by Yeo-Johnson transformation across all identified ROIs, then modeled by a GEE model with identity link function and an exchangeable working correlation structure that captures the feature’s dependency among ROIs within the same patient [12]. The *P*-values corresponding to the CR groups were then adjusted for multiple testing across all compared radiomic features by the Benjamini-Hochberg procedure [13]. *P*-values below 0.05, including adjusted *P*-values, were considered statistically significant. All statistical analyses were performed using R statistical software (R Foundation for Statistical Computing, version 4.2.0).

**REFERENCES**

1. Abramson JS, Palomba ML, Gordon LI, Lunning MA, Wang M, Arnason J, et al. Lisocabtagene maraleucel for patients with relapsed or refractory large B- cell lymphomas (TRANSCEND NHL 001): a multicenter seamless design study. Lancet. 2020;396(10254):839-52.

2. Harris PA, Taylor R, Minor BL, Elliott V, Fernandez M, O’Neal L, et al. The REDCap consortium: Building an international community of software platform partners. J Biomed Inform. 2019;95:103208.

3. Cheson BD, Fisher RI, Barrington SF, Cavalli F, Schwartz LH, Zucca E, et al. Recommendations for Initial Evaluation, Staging, and Response Assessment of Hodgkin and Non-Hodgkin Lymphoma: The Lugano Classification. J Clin Oncol. 2014;32(27):3059-67.

4. Lee DW, Santomasso BD, Locke FL, Ghobadi A, Turtle CJ, Brudno JN, et al. ASTCT Consensus Grading for Cytokine Release Syndrome and Neurologic Toxicity Associated with Immune Effector Cells. Biol Blood Marrow Transplant. 2019;25(4):625-38.

5. Kanoun S, Tal I, Berriolo-Riedinger A, Rossi C, Riedinger JM, Vrigneaud JM, et al. Influence of software tool and methodological aspects of total metabolic tumor volume calculation on baseline [18F]FDG PET to predict survival in Hodgkin lymphoma. PLoS One. 2015;10(10):e0140830.

6. Voltin CA, Gödel P, Beckmann L, Heger JM, Kobe C, Kutsch N, et al. Outcome Prediction in Patients With Large B-cell Lymphoma Undergoing Chimeric Antigen Receptor T-cell Therapy. Hemasphere. 2023;7(1):e817.

7. Barrington SF, Zwezerijnen BGJC, de Vet HCW, Heymans MW, Mikhaeel NG, Burggraaff CN, et al. Automated Segmentation of Baseline Metabolic Total Tumor Burden in Diffuse Large B-Cell Lymphoma: Which Method Is Most Successful? A Study on Behalf of the PETRA Consortium. J Nucl Med. 2021;62(3):332-7.

8. Kumar A, Burger IA, Zhang Z, Drill EN, Migliacci JC, Ng A, et al. Definition of bulky disease in early stage Hodgkin lymphoma in computed tomography era: prognostic significance of measurements in the coronal and transverse planes. Haematologica. 2016;101(10):1237-43.

9. Zwanenburg A, Vallières M, Abdalah MA, Aerts HJWL, Andrearczyk V, Apte A, et al. The image biomarker standardization initiative: standardized quantitative radiomics for high-throughput image-based phenotyping. Radiology. 2020;295(2):328-38.

10. Yip SSF, Parmar C, Kim J, Huynh E, Mak RH, Aerts HJWL. Impact of experimental design on PET radiomics in predicting somatic mutation status. Eur J Radiol. 2017;97:8-15.

11. Lausen B, Schumacher M. Maximally Selected Rank Statistics. Biometrics. 1992;48:73-85.

12. Yeo IK, Johnson RA. A new family of power transformations to improve normality or symmetry. Biometrika. 2000;87(4):954-9.

13. Benjamini Y, Hochberg Y. Controlling the false discovery rate: a practical and powerful approach to multiple testing. J R Stat Soc: Series B (Methological). 1995;57(1):289-300.
