# Supplementary material for: Conventional and novel [18F]FDG PET/CT features as predictors of CAR-T cell therapy outcome in large B-cell lymphoma
Source: J Hematol Oncol. 2024 Apr 23;17:21. doi: 10.1186/s13045-024-01540-x (PMC11035117; doi:10.1186/s13045-024-01540-x)
Supplement: Supplementary file 2 — Supplementary Material 2 [file 13045_2024_1540_MOESM2_ESM.docx]

**ADDITIONAL FILE 2**

**Supplementary Results**

**Patient characteristics and outcomes**

The cohort included 180 patients with LBCL, with a median age of 66 (range 20-86) years. The most common indications for CAR-T therapy were diffuse LBCL, not otherwise specified (n=136 [76%]); high-grade B-cell lymphoma with MYC and BCL2 and/or BCL 6 rearrangement (n=25 [14%]); and high-grade B-cell lymphoma, not otherwise specified (n=7 [4%]). Most patients (70%) had a Karnofsky performance status <90 prior to lymphodepletion and most (73%) received bridging therapy following apheresis. Tisa-cel and liso-cel (4-1BB costimulatory domain) were used in 52 (29%) and 35 (19%) cases, respectively, and axi-cel (CD28 costimulatory domain) was used in 93 (52%) cases. CRS and ICANS grade ≥2 were reported in 74 (41%) and 35 (19%) patients, respectively.

At 100 days after CAR-T cell infusion, 79% of patients were classified as responders: 58% showed CR, and 21% showed PR. The median OS was 24 months (95% CI, 17 – not reached), and the median PFS was 6.2 months (95% CI, 3.4 – 9.7), with a median follow-up period of 29 months (interquartile range [IQR], 18 – 40).

**PET features and laboratory biomarkers**

Overall, 341 PET/CT scans were evaluated, including 161 aph-PET and 180 car-PET scans. On aph-PET, the median values for SUVmax, MTV, and TLG were 19, 56 mL, and 427 mL, respectively; nodal conglomerates with a maximum diameter of 6-10 cm or >10 cm were found in 22 and 28 patients, respectively. On car-PET, the median values for SUVmax, MTV, and TLG were 15, 44 mL, and 338 mL, respectively; residual disease with a maximum diameter of 6-10 cm or >10 cm was found in 41 and 30 patients, respectively. Apheresis and infusion were approximately 5 weeks apart (median 36 days [IQR, 29-46]).

Inflammatory markers have been previously demonstrated to predict CAR-T therapy outcomes [1, 2]; therefore, we investigated correlations between PET imaging features and blood laboratory values of tumor burden (LDH) and inflammation (CRP, IL-6, IL-10, TNF-α, ferritin, fibrinogen, D-dimer) in all patients at pre-apheresis and/or pre-CAR-T cell infusion time points. Overall, markers of tumor burden and inflammation were moderately correlated with SUVmax, MTV and TLG in both aph-PET and car-PET scans.

**PET features and treatment toxicity**

In the univariable analysis, higher SUVmax and MTV at both aph-PET and car-PET were associated with a higher likelihood of grade ≥2 ICANS; the association of higher car-PET MTV with a higher likelihood of grade ≥2 CRS was marginally not significant (*P*=0.053). In the multivariable analysis, only car-PET MTV showed an association (OR 1.08 for 100-unit increase [95%CI 1.01-1.20], *P*=0.031) with CRS following adjustment for age, bridging therapy, pre-lymphodepletion LDH, and CAR-T product categorized by costimulatory domain. Thus, higher MTV at last disease assessment before lymphodepletion appears to be an independent risk factor for CRS, after adjusting for other clinical factors.

**PET features and clinical outcomes**

We hypothesized that PET features would (1) predict the likelihood of achieving CR, and (2) show associations with OS and PFS following CAR-T therapy. The factor most consistently associated with durable long-term remissions and survival following CAR-T cell therapy is the depth of initial response to treatment; hence, we focused on CR as our primary endpoint for radiomic analysis [3].

***Conventional features.*** Failure to acheive day 100 CR after CAR-T therapy was associated with higher aph-PET and car-PET SUVmax, MTV, and TLG, as well as pre-lymphodepletion LDH on univariable analysis. In a multivariable model adjusted for age, pre-lymphodepletion LDH, CAR-T costimulatory domain, and bridging therapy, the odds of non-CR were higher for those with a higher car-PET SUVmax (OR 1.72 for 10-unit increase [95% CI 1.24-2.43], *P*<0.001), whereas MTV lacked such an association.

***Novel features.*** Of 116 car-PET radiomic and segmentation features, 47 were associated with CR. In the GEE models adjusted for age, costimulatory factor, LDH, and transformed NHL, radiomic texture features, from feature groups such as gray-level dependence and run-length matrix, which are based on the distribution of interconnected voxels with identical or dependent gray levels, and shape-based features had significant differential expression between CR and non-CR ROIs (adjusted *P*<0.05). Thus, in addition to conventional PET metabolic features, more granular radiomic features describing intralesional metabolic heterogeneity beyond the perception of the human eye might be able to predict resistance to CAR-T therapy.

Shorter PFS was significantly associated with higher aph-PET MTV and TLG, as well as with higher car-PET SUVmax, MTV, TLG, and bulk on univariable Cox regression analysis. In the multivariable analysis, higher aph-PET MTV was significantly associated with shorter PFS (HR 1.11 for 10-unit increase [95% CI 1.05-1.17], *P*<0.001); higher car-PET MTV (HR 1.04 for 10-unit increase [95% CI 1.02-1.07], *P*<0.001) and elevated LDH (HR 1.59 [1.05-2.41], *P*=0.029) also remained significant predictors of PFS. Similarly, shorter OS was associated with higher SUVmax, MTV, TLG, and bulk category at both time points. In a multivariable Cox regression model, aph-PET MTV was associated with shorter OS (HR 1.14 for 100-unit increase [95% CI 1.07-1.21], *P*<0.001); higher car-PET MTV (HR 1.04 for 100-unit increase [95% CI 1.02-1.06], *P*<0.001) and elevated pre-lymphodepletion LDH (HR 2.65 [95%CI 1.59-4.41], *P*<0.001) also remained associated with shorter OS. Since MTV was consistently associated with worse outcome, we calculated an optimal threshold (MTV=24 mL) at car-PET to separate patients with favorable and unfavorable PFS.

Since MTV and LDH were significant predictors of PFS and OS on multivariable analysis, showing that both had independent prognostic relevance, we investigated their combined prognostic value, and observed that the combination of both parameters further separated high and low PFS and OS risk groups, as well as two intermediate risk groups. The threshold for MTV (cutoff of 24 mL) in this combination was chosen statistically, using maximally selected rank statistics, independently from LDH. Both parameters are easily accessible and may therefore prove useful in clinical practice.

**REFERENCES**

1. Locke FL, Ghobadi A, Jacobson CA, Miklos DB, Lekakis LJ, Oluwole OO, et al. Long-term safety and activity of axicabtagene ciloleucel in refractory large B-cell lymphoma (ZUMA-1): a single-arm, multicenter, phase 1-2 trial. Lancet Oncol. 2019;20(1):31-42.

2. Jain MD, Zhao H, Wang X, Atkins R, Menges M, Reid K, et al. Tumor interferon signaling and suppressive myeloid cells are associated with CAR T-cell failure in large B-cell lymphoma. Blood. 2021;137(19):2621-33.

3. Cappell KM, Kochenderfer JN. Long-term outcomes following CAR T cell therapy: what we know so far. Nat Rev Clin Oncol. 2023;29(6):359-71.
