# Supplementary material for: Conventional and novel [18F]FDG PET/CT features as predictors of CAR-T cell therapy outcome in large B-cell lymphoma
Source: J Hematol Oncol. 2024 Apr 23;17:21. doi: 10.1186/s13045-024-01540-x (PMC11035117; doi:10.1186/s13045-024-01540-x)
Supplement: Supplementary file 3 — Supplementary Material 3 [file 13045_2024_1540_MOESM3_ESM.docx]

**ADDITIONAL FILE 3**

**Supplementary Tables**

**Supplementary Table 1. Patient characteristics stratified by optimal car-PET metabolic tumor volume cutoff based on PFS.**

| **Characteristic** | **Overall, n=180^*^** | **MTV ≤24^**^, n=80** | **MTV >24, n=99** |
| --- | --- | --- | --- |
| Pre-CAR-T age, median (range), years | 66 (20 – 86) | 64 (20 – 84) | 67 (24 – 86) |
| Sex, n (%) |  |  |  |
| Male | 121 (67%) | 48 (60%) | 72 (73%) |
| Pre-CAR-T KPS (categorized), n (%) |  |  |  |
| <90 | 126 (70%) | 58 (73%) | 68 (69%) |
| Unknown | 1 | 0 | 1 |
| Diagnosis, n (%) |  |  |  |
| DLBCL NOS | 136 (76%) | 67 (84%) | 68 (69%) |
| HBCL w/ MYC, BCL2, BCL6 | 25 (14%) | 5 (6%) | 20 (20%) |
| PMBCL | 5 (3%) | 0 (0%) | 5 (5%) |
| HBCL, NOS | 7 (4%) | 3 (4%) | 4 (4%) |
| EBV-positive DLBCL | 4 (2%) | 2 (2.5%) | 2 (2%) |
| T-cell rich DLBCL | 3 (2%) | 3 (4%) | 0 (0%) |
| Transformed NHL, n (%) | 71 (40%) | 36 (45%) | 35 (36%) |
| Unknown | 1 | 0 | 1 |
| Aph-PET SUVmax, median (range) | 19 (0 – 48) | 15 (0 – 47) | 21 (0 – 48) |
| Unknown | 19 | 5 | 14 |
| Aph-PET MTV (mL), median (range) | 56 (0 – 2,278) | 12 (0 – 1,266) | 102 (0 – 2,278) |
| Unknown | 20 | 5 | 14 |
| Aph-PET TLG (mL), median (range) | 427 (0 – 38,426) | 76 (0 – 20,760) | 872 (0 – 38,426) |
| Unknown | 20 | 5 | 14 |
| Aph-PET max. diameter/bulk categories, n (%) |  |  |  |
| <6 cm | 130 (72%) | 65 (81%) | 64 (65%) |
| 6-10 cm | 22 (12%) | 7 (9%) | 15 (15%) |
| >10 cm | 28 (16%) | 8 (10%) | 20 (20%) |
| Car-PET SUVmax, median (range) | 15 (0 – 48) | 5 (0 – 44) | 22 (7 – 48) |
| Car-PET MTV (mL), median (range) | 44 (0 – 6,313) | 2 (0 – 24) | 167 (24 – 6,313) |
| Unkown | 1 | 0 | 0 |
| Car-PET TLG (mL), median (range) | 338 (0 – 52,051) | 8 (0 – 220) | 1,387 (153 – 52,051) |
| Unknown | 1 | 0 | 0 |
| Car-PET max. diameter/bulk categories, n (%) |  |  |  |
| <6 cm | 109 (61%) | 67 (84%) | 41 (41%) |
| 6-10 cm | 41 (23%) | 10 (12%) | 31 (31%) |
| >10 cm | 30 (17%) | 3 (4%) | 27 (27%) |
| LDH range pre-lymphodepletion, n (%) |  |  |  |
| Elevated | 81 (45%) | 16 (20%) | 65 (66%) |
| Pre-apheresis treatments (categorized), n (%) |  |  |  |
| ≤3 lines | 106 (60%) | 49 (62%) | 56 (57%) |
| 4-5 lines | 44 (25%) | 20 (25%) | 24 (24%) |
| ≥6 lines | 28 (16%) | 10 (13%) | 18 (18%) |
| Unknown | 2 | 1 | 1 |
| Primary refractory - pre-apheresis, n (%) | 64 (36%) | 21 (26%) | 43 (43%) |
| Bridging, n (%) | 132 (73%) | 58 (72.5%) | 73 (74%) |
| Pre-CAR-T disease response, n (%) |  |  |  |
| CR | 10 (6%) | 10 (12.5%) | 0 (0%) |
| PR | 35 (19%) | 28 (35%) | 6 (6%) |
| SD | 12 (7%) | 4 (5%) | 8 (8%) |
| PD | 123 (68%) | 38 (48%) | 85 (86%) |
| CAR-T product, n (%) |  |  |  |
| Axicabtagene ciloleucel | 93 (52%) | 41 (51%) | 51 (52%) |
| Tisagenlecleucel | 52 (29%) | 29 (36%) | 23 (23%) |
| Lisocabtagene maraleucel | 35 (19%) | 10 (12.5%) | 25 (25%) |
| CAR-T costimulatory domain, n (%) |  |  |  |
| CD28 | 93 (52%) | 41 (51%) | 51 (52%) |
| 41BB | 87 (49%) | 39 (49%) | 48 (48%) |

Abbreviations: Aph-PET = pre-leukapheresis PET scan; Car-PET = pre-CAR-T cell infusion PET scan; CR = complete response; DLBCL = diffuse large B-cell lymphoma; EBV = Epstein Barr Virus; HBCL = high-grade B-cell lymphoma; KPS = Karnofsky performance status; LDH = lactate dehydrogenase; MTV = metabolic tumor volume; NHL = non-Hodgkin lymphoma; NOS = not otherwise specified; PD = progressive disease; PMBCL = primary mediastinal B-cell lymphoma, PR = partial remission; SD = stable disease; SUVmax = maximum standardized uptake value; TLG = total lesion glycolysis. ^*^One patient with missing volume. ^**^An optimal MTV cutpoint of 24 mL was chosen based on maximally selected rank statistics.

**Supplementary Table 2. Association of PET features with grade 2 or higher cytokine release syndrome.**

| \|  \| **Univariable** \| \| \| **Multivariable aph-PET** \| \| \| **Multivariable car-PET** \| \| \| \| --- \| --- \| --- \| --- \| --- \| --- \| --- \| --- \| --- \| --- \| \| **Characteristic** \| **N** \| **OR (95% CI)** \| ***p*** \| **N** \| **OR (95% CI)** \| ***p*** \| **N** \| **OR (95% CI)** \| ***p*** \| \| Car-PET SUVmax^*^ \| 180 \| 0.95 (0.73-1.22) \| 0.68 \|  \|  \|  \| 179 \| 0.89 (0.65-1.21) \| 0.47 \| \| Car-PET MTV (mL)^**^ \| 179^Δ^ \| 1.06 (1.00-1.15) \| 0.053 \|  \|  \|  \| 179 \| 1.08 (1.01-1.20) \| 0.031 \| \| Pre-CAR-T age \| 180 \| 1.02 (1.00-1.04) \| 0.083 \| 160 \| 1.03 (1.01-1.06) \| 0.022 \| 179 \| 1.04 (1.01-1.07) \| 0.006 \| \| LDH range pre-lymphodepletion \| 180 \|  \| 0.48 \| 160 \|  \| 0.21 \| 179 \|  \| 0.22 \| \| normal \|  \| — \|  \|  \| — \|  \|  \| — \|  \| \| elevated \|  \| 0.81 (0.44-1.47) \|  \|  \| 0.63 (0.31-1.28) \|  \|  \| 0.63 (0.30-1.32) \|  \| \| CAR-T costimulatory domain \| 180 \|  \| 0.008 \| 160 \|  \| 0.015 \| 179 \|  \| <0.001 \| \| CD28 \|  \| — \|  \|  \| — \|  \|  \| — \|  \| \| 41BB \|  \| 0.44 (0.24-0.81) \|  \|  \| 0.40 (1.18-0.83) \|  \|  \| 0.32 (0.16-0.63) \|  \| \| Bridging \| 180 \|  \| 0.55 \| 160 \|  \| 0.71 \| 179 \|  \| 0.91 \| \| no \|  \| — \|  \|  \| — \|  \|  \| — \|  \| \| yes \|  \| 1.23 (0.63-2.45) \|  \|  \| 1.17 (0.53-2.67) \|  \|  \| 1.04 (0.50-2.18) \|  \| \| NHL transformation origin \| 179 \|  \| 0.26 \|  \|  \|  \|  \|  \|  \| \| de novo LBCL \|  \| — \|  \|  \|  \|  \|  \|  \|  \| \| transformed FL \|  \| 1.50 (0.75-2.99) \|  \|  \|  \|  \|  \|  \|  \| \| other primary \|  \| 1.93 (0.78-4.85) \|  \|  \|  \|  \|  \|  \|  \| \| Aph-PET SUVmax^*^ \| 161 \| 0.82 (0.60-1.10) \| 0.18 \| 160 \| 0.74 (0.51-1.05) \| 0.10 \|  \|  \|  \| \| Aph-PET MTV (mL)^**^ \| 160^Δ^ \| 1.03 (0.94-1.14) \| 0.47 \| 160 \| 1.05 (0.94-1.18) \| 0.35 \|  \|  \|  \| \| Aph-PET TLG (mL)^***^ \| 160^Δ^ \| 1.04 (0.97-1.14) \| 0.26 \|  \|  \|  \|  \|  \|  \| \| Aph-PET max. diameter/bulk categories \| 180 \|  \| 0.14 \|  \|  \|  \|  \|  \|  \| \| <6 cm \|  \| — \|  \|  \|  \|  \|  \|  \|  \| \| 6-10 cm \|  \| 0.72 (0.26-1.84) \|  \|  \|  \|  \|  \|  \|  \| \| >10 cm \|  \| 2.07 (0.91-4.81) \|  \|  \|  \|  \|  \|  \|  \| \| Car-PET TLG (mL)^***^ \| 179^Δ^ \| 1.08 (1.01-1.18) \| 0.024 \|  \|  \|  \|  \|  \|  \| \| Car-PET max. diameter/bulk categories \| 180 \|  \| 0.014 \|  \|  \|  \|  \|  \|  \| \| <6 cm \|  \| — \|  \|  \|  \|  \|  \|  \|  \| \| 6-10 cm \|  \| 0.64 (0.28-1.35) \|  \|  \|  \|  \|  \|  \|  \| \| >10 cm \|  \| 2.65 (1.17-6.29) \|  \|  \|  \|  \|  \|  \|  \|   Abbreviations: Aph-PET = pre-leukapheresis PET scan; Car-PET = pre-CAR-T cell infusion PET scan; CI = confidence intervals; FL = follicular lymphoma; LBCL = large B-cell lymphoma; LDH = lactate dehydrogenase; MTV = metabolic tumor volume; NHL = non-Hodgkin lymphoma; OR = odds ratio; SUVmax = maximum standardized uptake value; TLG = total lesion glycolysis. ^Δ^ One patient with missing volume. * For 10 units. ** For 100 units. *** For 1000 units.  **Supplementary Table 3. Association of PET features with grade 2 or higher immune effector cell-associated neurotoxicity syndrome.**   \| \|  \| **Univariable** \| \| \| **Multivariable aph-PET** \| \| \| **Multivariable car-PET** \| \| \| \| --- \| --- \| --- \| --- \| --- \| --- \| --- \| --- \| --- \| --- \| \| **Characteristic** \| **N** \| **OR (95% CI)** \| ***p*** \| **N** \| **OR (95% CI)** \| ***p*** \| **N** \| **OR (95% CI)** \| ***p*** \| \| Aph-PET SUVmax* \| 161 \| 1.62 (1.12-2.42) \| 0.011 \| 160 \| 1.49 (0.95-2.36) \| 0.087 \|  \|  \|  \| \| Aph-PET MTV (mL)** \| 160^Δ^ \| 1.11 (1.00-1.23) \| 0.040 \| 160 \| 1.03 (0.92-1.15) \| 0.59 \|  \|  \|  \| \| Car-PET SUVmax* \| 180 \| 1.46 (1.07-2.03) \| 0.018 \|  \|  \|  \| 179 \| 1.34 (0.92-1.96) \| 0.13 \| \| Car-PET MTV (mL)** \| 179^Δ^ \| 1.06 (1.00-1.14) \| 0.042 \|  \|  \|  \| 179 \| 1.04 (0.98-1.12) \| 0.21 \| \| Pre-CAR-T age \| 180 \| 1.02 (0.99-1.05) \| 0.30 \| 160 \| 1.03 (0.99-1.07) \| 0.13 \| 179 \| 1.03 (1.00-1.07) \| 0.058 \| \| LDH range pre-LD \| 180 \|  \| 0.11 \| 160 \|  \| 0.26 \| 179 \|  \| 0.64 \| \| normal \|  \| — \|  \|  \| — \|  \|  \| — \|  \| \| elevated \|  \| 1.84 (0.87-3.93) \|  \|  \| 1.66 (0.69-4.03) \|  \|  \| 1.24 (0.50-3.04) \|  \| \| CAR-T costimulatory domain \| 180 \|  \| <0.001 \| 160 \|  \| 0.006 \| 179 \|  \| <0.001 \| \| CD28 \|  \| — \|  \|  \| — \|  \|  \| — \|  \| \| 41BB \|  \| 0.25 (0.10-0.56) \|  \|  \| 0.25 (0.09-0.65) \|  \|  \| 0.18 (0.07-0.45) \|  \| \| NHL transformation origin \| 179 \|  \| 0.73 \|  \|  \|  \|  \|  \|  \| \| de novo \|  \| __ \|  \|  \|  \|  \|  \|  \|  \| \| tFL \|  \| 1.39 (0.59-3.18) \|  \|  \|  \|  \|  \|  \|  \| \| other primary \|  \| 0.99 (0.26-3.00) \|  \|  \|  \|  \|  \|  \|  \| \| Bridging \| 180 \|  \| 0.57 \| 160 \|  \| 0.33 \| 179 \|  \| 0.85 \| \| no \|  \| — \|  \|  \| — \|  \|  \| — \|  \| \| yes \|  \| 1.29 (0.56-3.24) \|  \|  \| 0.60 (0.21-1.72) \|  \|  \| 1.10 (0.44-2.94) \|  \| \| Aph-PET TLG (mL)*** \| 160^Δ^ \| 1.10 (1.01-1.21) \| 0.024 \|  \|  \|  \|  \|  \|  \| \| Aph-PET max. diameter/bulk categories \| 180 \|  \| 0.21 \|  \|  \|  \|  \|  \|  \| \| <6 cm \|  \| — \|  \|  \|  \|  \|  \|  \|  \| \| 6-10 cm \|  \| 1.09 (0.29-3.27) \|  \|  \|  \|  \|  \|  \|  \| \| >10 cm \|  \| 2.33 (0.90-5.73) \|  \|  \|  \|  \|  \|  \|  \| \| Car-PET TLG (mL)*** \| 179^Δ^ \| 1.09 (1.02-1.19) \| 0.012 \|  \|  \|  \|  \|  \|  \| \| Car-PET max. diameter/bulk categories \| 180 \|  \| 0.14 \|  \|  \|  \|  \|  \|  \| \| <6 cm \|  \| — \|  \|  \|  \|  \|  \|  \|  \| \| 6-10 cm \|  \| 1.04 (0.38-2.62) \|  \|  \|  \|  \|  \|  \|  \| \| >10 cm \|  \| 2.53 (0.99-6.25) \|  \|  \|  \|  \|  \|  \|  \| \|  \|  \|  \|  \|  \| \| --- \| --- \| --- \| --- \| --- \| --- \| --- \| --- \| --- \| --- \| --- \| --- \| --- \| --- \| --- \| --- \| --- \| --- \| --- \| --- \| --- \| --- \| --- \| --- \| --- \| --- \| --- \| --- \| --- \| --- \| --- \| --- \| --- \| --- \| --- \| --- \| --- \| --- \| --- \| --- \| --- \| --- \| --- \| --- \| --- \| --- \| --- \| --- \| --- \| --- \| --- \| --- \| --- \| --- \| --- \| --- \| --- \| --- \| --- \| --- \| --- \| --- \| --- \| --- \| --- \| --- \| --- \| --- \| --- \| --- \| --- \| --- \| --- \| --- \| --- \| --- \| --- \| --- \| --- \| --- \| --- \| --- \| --- \| --- \| --- \| --- \| --- \| --- \| --- \| --- \| --- \| --- \| --- \| --- \| --- \| --- \| --- \| --- \| --- \| --- \| --- \| --- \| --- \| --- \| --- \| --- \| --- \| --- \| --- \| --- \| --- \| --- \| --- \| --- \| --- \| --- \| --- \| --- \| --- \| --- \| --- \| --- \| --- \| --- \| --- \| --- \| --- \| --- \| --- \| --- \| --- \| --- \| --- \| --- \| --- \| --- \| --- \| --- \| --- \| --- \| --- \| --- \| --- \| --- \| --- \| --- \| --- \| --- \| --- \| --- \| --- \| --- \| --- \| --- \| --- \| --- \| --- \| --- \| --- \| --- \| --- \| --- \| --- \| --- \| --- \| --- \| --- \| --- \| --- \| --- \| --- \| --- \| --- \| --- \| --- \| --- \| --- \| --- \| --- \| --- \| --- \| --- \| --- \| --- \| --- \| --- \| --- \| --- \| --- \| --- \| --- \| --- \| --- \| --- \| --- \| --- \| --- \| --- \| --- \| --- \| --- \| --- \| --- \| --- \| --- \| --- \| --- \| --- \| --- \| --- \| --- \| --- \| --- \| --- \| --- \| --- \| --- \| --- \| --- \| --- \| --- \| --- \| --- \| --- \| --- \| --- \| --- \| --- \| --- \| --- \| --- \| --- \| --- \| --- \| --- \| --- \| --- \| --- \| --- \| --- \| --- \| --- \| --- \| --- \| --- \| --- \| --- \| --- \| --- \| --- \| --- \| --- \| --- \| --- \| --- \| --- \| --- \| --- \| --- \| --- \| --- \| --- \| --- \| --- \| --- \| --- \| --- \| --- \| --- \| --- \| --- \| --- \| --- \| --- \| --- \| --- \| --- \| --- \| --- \| --- \| --- \| --- \| --- \| --- \| --- \| --- \| --- \| --- \| --- \| --- \| --- \| --- \| --- \| --- \| --- \| --- \| --- \| --- \| --- \| --- \| --- \| --- \| --- \| --- \| --- \| --- \|   Abbreviations: Aph-PET = pre-leukapheresis PET scan; Car-PET = pre-CAR-T cell infusion PET scan; CI = confidence intervals; LD = lymphodepletion; LDH = lactate dehydrogenase; MTV = metabolic tumor volume; NHL = non-Hodgkin lymphoma; OR = odds ratio; SUVmax = maximum standardized uptake value; tFL = transformed follicular lymphoma; TLG = total lesion glycolysis. ^Δ^ One patient with missing volume. * For 10 units. ** For 100 units. *** For 1000 units.  **Supplementary Table 4. PET features and complete response.**   \|  \| **Univariable** \| \| \| **Multivariable aph-PET** \| \| \| **Multivariable car-PET** \| \| \| \| --- \| --- \| --- \| --- \| --- \| --- \| --- \| --- \| --- \| --- \| \| **Characteristic** \| **N** \| **OR (95% CI)** \| ***p*** \| **N** \| **OR (95% CI)** \| ***p*** \| **N** \| **OR (95% CI)** \| ***p*** \| \| Car-PET SUVmax* \| 177 \| 1.93 (1.45-2.61) \| <0.001 \|  \|  \|  \| 177 \| 1.72 (1.24-2.43) \| <0.001 \| \| Car-PET MTV (mL)** \| 177 \| 1.23 (1.10-1.42) \| <0.001 \|  \|  \|  \| 177 \| 1.09 (1.0-1.26) \| 0.072 \| \| Pre-CAR-T age \| 177 \| 1.00 (0.98-1.02) \| 0.95 \| 158 \| 1.00 (0.97-1.02) \| 0.72 \| 177 \| 0.99 (0.97-1.92) \| 0.60 \| \| LDH range pre-LD \| 177 \|  \| <0.001 \| 158 \|  \| 0.009 \| 177 \|  \| 0.39 \| \| normal \|  \| — \|  \|  \| — \|  \|  \| — \|  \| \| elevated \|  \| 2.85 (1.55-5.33) \|  \|  \| 2.51 (1.26-5.03) \|  \|  \| 1.38 (0.66-2.87) \|  \| \| CAR-T costimulatory domain \| 177 \|  \| 0.66 \| 158 \|  \| 0.21 \| 177 \|  \| 0.35 \| \| CD28 \|  \| — \|  \|  \| — \|  \|  \| — \|  \| \| 41BB \|  \| 1.15 (0.63-2.09) \|  \|  \| 1.62 (0.77-3.46) \|  \|  \| 1.38 (0.70-2.76) \|  \| \| Bridging \| 177 \|  \| 0.079 \| 158 \|  \| 0.53 \| 177 \|  \| 0.10 \| \| no \|  \| — \|  \|  \| — \|  \|  \| — \|  \| \| yes \|  \| 1.85 (0.93-3.82) \|  \|  \| 1.30 (0.57-3.02) \|  \|  \| 1.88 (0.88-4.17) \|  \| \| NHL tf origin \| 176 \|  \| 0.32 \|  \|  \|  \|  \|  \|  \| \| de novo \|  \| — \|  \|  \|  \|  \|  \|  \|  \| \| tFL \|  \| 0.71 (0.35-1.42) \|  \|  \|  \|  \|  \|  \|  \| \| other primary \|  \| 0.52 (0.19-1.32) \|  \|  \|  \|  \|  \|  \|  \| \| Aph-PET SUVmax* \| 158 \| 1.59 (1.17-2.20) \| 0.003 \| 158 \| 1.40 (0.99-2.03) \| 0.064 \|  \|  \|  \| \| Aph-PET MTV (mL)** \| 158 \| 1.13 (1.02-1.28) \| 0.021 \| 158 \| 1.07 (0.95-1.23) \| 0.30 \|  \|  \|  \| \| Aph-PET TLG (mL)*** \| 158 \| 1.10 (1.01-1.24) \| 0.036 \|  \|  \|  \|  \|  \|  \| \| Aph-PET max. diameter/bulk categories \| 177 \|  \| 0.11 \|  \|  \|  \|  \|  \|  \| \| <6 cm \|  \| — \|  \|  \|  \|  \|  \|  \|  \| \| 6-10 cm \|  \| 1.69 (0.67-4.23) \|  \|  \|  \|  \|  \|  \|  \| \| >10 cm \|  \| 2.30 (0.98-5.54) \|  \|  \|  \|  \|  \|  \|  \| \| Car-PET TLG (mL)*** \| 177 \| 1.22 (1.09-1.40) \| <0.001 \|  \|  \|  \|  \|  \|  \| \| Car-PET max. diameter/bulk categories \| 177 \|  \| <0.001 \|  \|  \|  \|  \|  \|  \| \| <6 cm \|  \| — \|  \|  \|  \|  \|  \|  \|  \| \| 6-10 cm \|  \| 3.03 (1.45-6.45) \|  \|  \|  \|  \|  \|  \|  \| \| >10 cm \|  \| 5.01 (2.10-12.8) \|  \|  \|  \|  \|  \|  \|  \| |  |  |  |  |  |
| --- | --- | --- | --- | --- | --- | --- | --- | --- | --- | --- | --- | --- | --- | --- | --- | --- | --- | --- | --- | --- | --- | --- | --- | --- | --- | --- | --- | --- | --- | --- | --- | --- | --- | --- | --- | --- | --- | --- | --- | --- | --- | --- | --- | --- | --- | --- | --- | --- | --- | --- | --- | --- | --- | --- | --- | --- | --- | --- | --- | --- | --- | --- | --- | --- | --- | --- | --- | --- | --- | --- | --- | --- | --- | --- | --- | --- | --- | --- | --- | --- | --- | --- | --- | --- | --- | --- | --- | --- | --- | --- | --- | --- | --- | --- | --- | --- | --- | --- | --- | --- | --- | --- | --- | --- | --- | --- | --- | --- | --- | --- | --- | --- | --- | --- | --- | --- | --- | --- | --- | --- | --- | --- | --- | --- | --- | --- | --- | --- | --- | --- | --- | --- | --- | --- | --- | --- | --- | --- | --- | --- | --- | --- | --- | --- | --- | --- | --- | --- | --- | --- | --- | --- | --- | --- | --- | --- | --- | --- | --- | --- | --- | --- | --- | --- | --- | --- | --- | --- | --- | --- | --- | --- | --- | --- | --- | --- | --- | --- | --- | --- | --- | --- | --- | --- | --- | --- | --- | --- | --- | --- | --- | --- | --- | --- | --- | --- | --- | --- | --- | --- | --- | --- | --- | --- | --- | --- | --- | --- | --- | --- | --- | --- | --- | --- | --- | --- | --- | --- | --- | --- | --- | --- | --- | --- | --- | --- | --- | --- | --- | --- | --- | --- | --- | --- | --- | --- | --- | --- | --- | --- | --- | --- | --- | --- | --- | --- | --- | --- | --- | --- | --- | --- | --- | --- | --- | --- | --- | --- | --- | --- | --- | --- | --- | --- | --- | --- | --- | --- | --- | --- | --- | --- | --- | --- | --- | --- | --- | --- | --- | --- | --- | --- | --- | --- | --- | --- | --- | --- | --- | --- | --- | --- | --- | --- | --- | --- | --- | --- | --- | --- | --- | --- | --- | --- | --- | --- | --- | --- | --- | --- | --- | --- | --- | --- | --- | --- | --- | --- | --- | --- | --- | --- | --- | --- | --- | --- | --- | --- | --- | --- | --- | --- | --- | --- | --- | --- | --- | --- | --- | --- | --- | --- | --- | --- | --- | --- | --- | --- | --- | --- | --- | --- | --- | --- | --- | --- | --- | --- | --- | --- | --- | --- | --- | --- | --- | --- | --- | --- | --- | --- | --- | --- | --- | --- | --- | --- | --- | --- | --- | --- | --- | --- | --- | --- | --- | --- | --- | --- | --- | --- | --- | --- | --- | --- | --- | --- | --- | --- | --- | --- | --- | --- | --- | --- | --- | --- | --- | --- | --- | --- | --- | --- | --- | --- | --- | --- | --- | --- | --- | --- | --- | --- | --- | --- | --- | --- | --- | --- | --- | --- | --- | --- | --- | --- | --- | --- | --- | --- | --- | --- | --- | --- | --- | --- | --- | --- | --- | --- | --- | --- | --- | --- | --- | --- | --- | --- | --- | --- | --- | --- | --- | --- | --- | --- | --- | --- | --- | --- | --- | --- | --- | --- | --- | --- | --- | --- | --- | --- | --- | --- | --- | --- | --- | --- | --- | --- | --- | --- | --- | --- | --- | --- | --- | --- | --- | --- | --- | --- | --- | --- | --- | --- | --- | --- | --- | --- | --- | --- | --- | --- | --- | --- | --- | --- | --- | --- | --- | --- | --- | --- | --- | --- | --- | --- | --- | --- | --- | --- | --- | --- | --- | --- | --- | --- | --- | --- | --- | --- | --- | --- | --- | --- | --- | --- | --- | --- | --- | --- | --- | --- | --- | --- | --- | --- | --- | --- | --- | --- | --- | --- | --- | --- | --- | --- | --- | --- | --- | --- | --- | --- | --- | --- | --- | --- | --- | --- | --- | --- | --- | --- | --- | --- | --- | --- | --- | --- | --- | --- | --- | --- | --- | --- | --- | --- | --- | --- | --- | --- | --- | --- | --- | --- | --- | --- | --- | --- | --- | --- | --- | --- | --- | --- | --- | --- | --- | --- | --- | --- | --- | --- | --- | --- | --- | --- | --- | --- | --- | --- | --- | --- | --- | --- | --- | --- | --- | --- | --- | --- | --- | --- | --- | --- | --- | --- | --- | --- | --- | --- | --- | --- | --- | --- | --- | --- | --- | --- | --- | --- | --- | --- | --- | --- | --- | --- | --- | --- | --- | --- | --- | --- | --- | --- | --- | --- | --- | --- | --- | --- | --- | --- | --- | --- | --- | --- | --- | --- | --- | --- | --- | --- | --- | --- | --- | --- | --- | --- | --- | --- | --- | --- | --- | --- | --- | --- | --- | --- | --- | --- | --- | --- | --- | --- | --- | --- | --- | --- | --- | --- | --- | --- | --- | --- | --- | --- | --- | --- | --- | --- | --- | --- | --- | --- | --- | --- | --- | --- | --- | --- | --- | --- | --- | --- | --- | --- | --- | --- | --- | --- | --- | --- | --- | --- | --- | --- | --- | --- | --- | --- | --- | --- | --- | --- | --- | --- | --- | --- | --- | --- | --- | --- | --- | --- | --- | --- | --- | --- | --- | --- | --- | --- | --- | --- | --- | --- | --- | --- | --- | --- | --- | --- | --- | --- | --- | --- | --- | --- | --- | --- | --- | --- | --- | --- | --- | --- | --- | --- | --- | --- | --- | --- | --- | --- | --- | --- | --- | --- | --- | --- | --- | --- | --- | --- | --- | --- | --- | --- | --- | --- | --- | --- | --- | --- | --- | --- | --- | --- | --- | --- | --- | --- | --- | --- | --- | --- | --- | --- | --- | --- | --- | --- | --- | --- | --- | --- | --- | --- | --- | --- | --- | --- | --- | --- | --- | --- | --- | --- | --- | --- | --- | --- | --- | --- | --- | --- | --- | --- | --- | --- | --- | --- | --- | --- | --- | --- | --- | --- | --- | --- | --- | --- | --- | --- | --- | --- | --- | --- | --- | --- | --- | --- | --- | --- | --- | --- | --- | --- | --- | --- | --- | --- | --- |

Note: OR > 1 is associated with odds of not achieving CR. CR by day 100 information is missing for three patients. Abbreviations: Aph-PET = pre-leukapheresis PET scan; Car-PET = pre-CAR-T cell infusion PET scan; CI = confidence intervals; CR = complete response; LBCL = large B-cell lymphoma; LD = lymphodepletion; LDH = lactate dehydrogenase; MTV = metabolic tumor volume; NHL = non-Hodgkin lymphoma; OR = odds ratio; SUVmax = maximum standardized uptake value; tf = transformation; tFL = transformed follicular lymphoma; TLG = total lesion glycolysis. * For 10 units. ** For 100 units. *** For 1000 units.

**Supplementary Table 5. PET features and progression-free survival.**

|  | **Univariable** | | | **Multivariable aph-PET** | | | **Multivariable car-PET** | | |
| --- | --- | --- | --- | --- | --- | --- | --- | --- | --- |
| **Characteristic** | **N** | **HR (95% CI)** | ***p*** | **N** | **HR (95% CI)** | ***p*** | **N** | **HR (95% CI)** | ***p*** |
| Car-PET SUVmax* | 180 | 1.28 (1.10-1.47) | 0.001 |  |  |  | 179 | 1.12 (0.95-1.33) | 0.16 |
| Car-PET MTV (mL)** | 179^Δ^ | 1.05 (1.03-1.07) | <0.001 |  |  |  | 179 | 1.04 (1.02-1.07) | <0.001 |
| Pre-CAR-T age | 180 | 1.01 (0.99-1.02) | 0.35 | 160 | 0.99 (0.98-1.01) | 0.44 | 179 | 1.00 (0.99-1.01) | 0.91 |
| LDH range pre-LD | 180 |  | <0.001 | 160 |  | <0.001 | 179 |  | 0.029 |
| normal |  | — |  |  | — |  |  | — |  |
| elevated |  | 2.05 (1.43-2.93) |  |  | 2.10 (1.41-3.14) |  |  | 1.59 (1.05-2.41) |  |
| CAR-T costimulatory domain | 180 |  | 0.14 | 160 |  | 0.020 | 179 |  | 0.074 |
| CD28 |  | — |  |  | — |  |  | — |  |
| 41BB |  | 1.30 (0.91-1.87) |  |  | 1.67 (1.08-2.57) |  |  | 1.42 (0.97-2.07) |  |
| Aph-PET SUVmax* | 161 | 1.08 (0.91-1.27) | 0.38 | 160 | 0.91 (0.74-1.10) | 0.33 |  |  |  |
| Aph-PET MTV** | 160^Δ^ | 1.09 (1.04-1.14) | 0.001 | 160 | 1.11 (1.05-1.17) | <0.001 |  |  |  |
| NHL tf origin | 179 |  | 0.28 |  |  |  |  |  |  |
| de novo |  | — |  |  |  |  |  |  |  |
| tFL |  | 0.77 (0.50-1.16) |  |  |  |  |  |  |  |
| other primary |  | 0.70 (0.40-1.24) |  |  |  |  |  |  |  |
| Bridging | 180 |  | 0.41 |  |  |  |  |  |  |
| no |  | — |  |  |  |  |  |  |  |
| yes |  | 1.18 (0.79-1.77) |  |  |  |  |  |  |  |
| Aph-PET TLG (mL)*** | 160^Δ^ | 1.06 (1.03-1.10) | 0.004 |  |  |  |  |  |  |
| Aph-PET max. diameter/bulk categories | 180 |  | 0.16 |  |  |  |  |  |  |
| <6 cm |  | — |  |  |  |  |  |  |  |
| 6-10 cm |  | 1.28 (0.74-2.21) |  |  |  |  |  |  |  |
| >10 cm |  | 1.60 (0.99-2.59) |  |  |  |  |  |  |  |
| Car-PET TLG (mL)*** | 179^Δ^ | 1.06 (1.04-1.08) | <0.001 |  |  |  |  |  |  |
| Car-PET max. diameter/bulk categories^+^ | 180 |  | <0.001 |  |  |  |  |  |  |
| <6 cm |  | — |  |  |  |  |  |  |  |
| 6-10 cm |  | 1.74 (1.13-2.67) |  |  |  |  |  |  |  |
| >10 cm |  | 2.56 (1.58-4.13) |  |  |  |  |  |  |  |

Note: multivariable analysis stratified by bridging status. Abbreviations: Aph-PET = pre-leukapheresis PET scan; Car-PET = pre-CAR-T cell infusion PET scan; CI = confidence intervals; HR = hazard ratio; LD = lymphodepletion; LDH = lactate dehydrogenase; MTV = metabolic tumor volume; NHL = non-Hodgkin lymphoma; SUVmax = maximum standardized uptake value; tFL = transformed follicular lymphoma; tf = transformation; TLG = total lesion glycolysis. ^Δ^ One patient with missing volume. * For 10 units. ** For 100 units. *** For 1000 units. ^+^Disease bulk was not included in the multivariable analysis to avoid collinearity with MTV.

**Supplementary Table 6. PET features and overall survival.**

|  | **Univariable** | | | **Multivariable aph-PET** | | | **Multivariable car-PET** | | |
| --- | --- | --- | --- | --- | --- | --- | --- | --- | --- |
| **Characteristic** | **N** | **HR (95% CI)** | ***p*** | **N** | **HR (95% CI)** | ***p*** | **N** | **HR (95% CI)** | ***p*** |
| Car-PET SUVmax* | 180 | 1.33 (1.12-1.57) | 0.001 |  |  |  | 179 | 1.09 (0.90-1.32) | 0.37 |
| Car-PET MTV (mL)** | 179^Δ^ | 1.05 (1.04-1.07) | <0.001 |  |  |  | 179 | 1.04 (1.02-1.06) | <0.001 |
| Pre-CAR-T age | 180 | 1.01 (1.00-1.03) | 0.14 | 160 | 1.00 (0.99-1.02) | 0.70 | 179 | 1.01 (0.99-1.03) | 0.35 |
| LDH range pre-lymphodepletion | 180 |  | <0.001 | 160 |  | <0.001 | 179 |  | <0.001 |
| normal |  | — |  |  | — |  |  | — |  |
| elevated |  | 3.37 (2.14-5.30) |  |  | 3.47 (2.09-5.76) |  |  | 2.65 (1.59-4.41) |  |
| CAR-T costimulatory domain | 180 |  | 0.32 | 160 |  | 0.14 | 179 |  | 0.19 |
| CD28 |  | — |  |  | — |  |  | — |  |
| 41BB |  | 1.24 (0.81-1.91) |  |  | 1.47 (0.88-2.45) |  |  | 1.35 (0.86-2.12) |  |
| Aph-PET SUVmax* | 161 | 1.24 (1.02-1.52) | 0.036 | 160 | 0.91 (0.71-1.16) | 0.44 |  |  |  |
| Aph-PET MTV (mL)** | 160^Δ^ | 1.13 (1.08-1.19) | <0.001 | 160 | 1.14 (1.07-1.21) | <0.001 |  |  |  |
| NHL tf origin | 179 |  | 0.061 |  |  |  |  |  |  |
| de novo |  | — |  |  |  |  |  |  |  |
| tFL |  | 0.79 (0.48-1.30) |  |  |  |  |  |  |  |
| other primary |  | 0.41 (0.18-0.95) |  |  |  |  |  |  |  |
| Bridging | 180 |  | 0.10 |  |  |  |  |  |  |
| no |  | — |  |  |  |  |  |  |  |
| yes |  | 1.51 (0.91-2.52) |  |  |  |  |  |  |  |
| Aph-PET TLG (mL)*** | 160^Δ^ | 1.10 (1.06-1.14) | <0.001 |  |  |  |  |  |  |
| Aph-PET max. diameter/bulk categories | 180 |  | 0.029 |  |  |  |  |  |  |
| <6 cm |  | — |  |  |  |  |  |  |  |
| 6-10 cm |  | 0.98 (0.49-1.98) |  |  |  |  |  |  |  |
| >10 cm |  | 2.14 (1.26-3.64) |  |  |  |  |  |  |  |
| Car-PET TLG (mL)*** | 179^Δ^ | 1.06 (1.04-1.09) | <0.001 |  |  |  |  |  |  |
| Car-PET max. diameter/bulk categories | 180 |  | <0.001 |  |  |  |  |  |  |
| <6 cm |  | — |  |  |  |  |  |  |  |
| 6-10 cm |  | 1.78 (1.06-2.96) |  |  |  |  |  |  |  |
| >10 cm |  | 3.27 (1.91-5.61) |  |  |  |  |  |  |  |

Note: multivariable analysis stratified by bridging status. Abbreviations: Aph-PET = pre-leukapheresis PET scan; Car-PET = pre-CAR-T cell infusion PET scan; CI = confidence intervals; HR = hazard ratio; LDH = lactate dehydrogenase; MTV = metabolic tumor volume; NHL = non-Hodgkin lymphoma; SUVmax = maximum standardized uptake value; tf = transformation; tFL = transformed follicular lymphoma; TLG = total lesion glycolysis. ^Δ^ One patient with missing volume. * For 10 units. ** For 100 units. *** For 1000 units.

**Supplementary Table 7. Radiomic features significantly different between CR and non-CR ROIs.**

| **Feature** | **Mean scaled difference between CR and non-CR groups at ROI level** | **Adjusted P-value** |
| --- | --- | --- |
| Dependence Variance | -0.39 | 0.0077 |
| Least Axis Length | -0.43 | 0.0077 |
| Large Dependence High Gray Level Emphasis | -0.39 | 0.0077 |
| Energy | -0.38 | 0.0077 |
| Large Dependence Emphasis | -0.39 | 0.0077 |
| Large Dependence Low Gray Level Emphasis | -0.39 | 0.0077 |
| Gray Level Non Uniformity | -0.36 | 0.0077 |
| Total Energy | -0.38 | 0.0077 |
| Run Entropy | -0.36 | 0.0077 |
| Dependence Entropy | -0.35 | 0.0077 |
| Large Area High Gray Level Emphasis | -0.37 | 0.0077 |
| Mean Volume | -0.41 | 0.0077 |
| Large Area Emphasis | -0.37 | 0.0077 |
| Large Area Low Gray Level Emphasis | -0.37 | 0.0077 |
| Gray Level Non Uniformity | -0.35 | 0.0077 |
| Zone Percentage | 0.37 | 0.0077 |
| Voxel Volume | -0.36 | 0.0077 |
| Volume | -0.38 | 0.0083 |
| Maximum 2D Diameter Row | -0.34 | 0.0086 |
| Surface Area | -0.35 | 0.0086 |
| Dependence Non Uniformity Normalized | 0.34 | 0.0090 |
| Run Variance | -0.33 | 0.0096 |
| Flatness | -0.30 | 0.011 |
| Run Length Non Uniformity | -0.32 | 0.011 |
| Run Length Non Uniformity Normalized | 0.33 | 0.011 |
| Dependence Non Uniformity | -0.29 | 0.011 |
| Maximum 2D Diameter Slice | -0.31 | 0.013 |
| Minor Axis Length | -0.32 | 0.014 |
| Surface Volume Ratio | 0.30 | 0.014 |
| Long Run High Gray Level Emphasis | -0.31 | 0.018 |
| Mean Q-PET | -0.39 | 0.018 |
| Maximum 2D Diameter Column | -0.29 | 0.018 |
| Mean Wahl | -0.39 | 0.018 |
| Long Run Emphasis | -0.31 | 0.018 |
| Long Run Low Gray Level Emphasis | -0.31 | 0.018 |
| Small Dependence Low Gray Level Emphasis | -0.34 | 0.018 |
| Maximum 3D Diameter | -0.27 | 0.018 |
| SUVpeak | -0.38 | 0.018 |
| SUVmean | -0.38 | 0.018 |
| Small Dependence Emphasis | 0.34 | 0.018 |
| Run Percentage | 0.29 | 0.020 |
| Small Dependence High Gray Level Emphasis | 0.32 | 0.022 |
| SUVqPeak | -0.37 | 0.023 |
| Short Run Low Gray Level Emphasis | 0.26 | 0.037 |
| SUVmax | -0.35 | 0.037 |
| Major Axis Length | -0.23 | 0.046 |
| Range | -0.33 | 0.049 |

Note: Abbreviations: CR = complete response; ROI = region of interest; SUVmax = maximum standardized uptake value.
