# Supplementary material for: Conventional and novel [18F]FDG PET/CT features as predictors of CAR-T cell therapy outcome in large B-cell lymphoma
Source: J Hematol Oncol. 2024 Apr 23;17:21. doi: 10.1186/s13045-024-01540-x (PMC11035117; doi:10.1186/s13045-024-01540-x)

**ADDITIONAL FILE 4**

**Supplementary Figures**

**Supplementary Figure 1. CONSORT diagram.** Note: * Car-PET reflects scans performed at the latest assessment before CAR-T infusion; the majority of these scans were from imaging performed after apheresis (n=158). Twenty-two patients did not have post-apheresis imaging and the pre-apheresis imaging was used as last assessment. This population had shorter time from apheresis to infusion compared to the main population, and fewer patients receiving bridging. Abbreviation: LBCL = large B cell lymphoma.


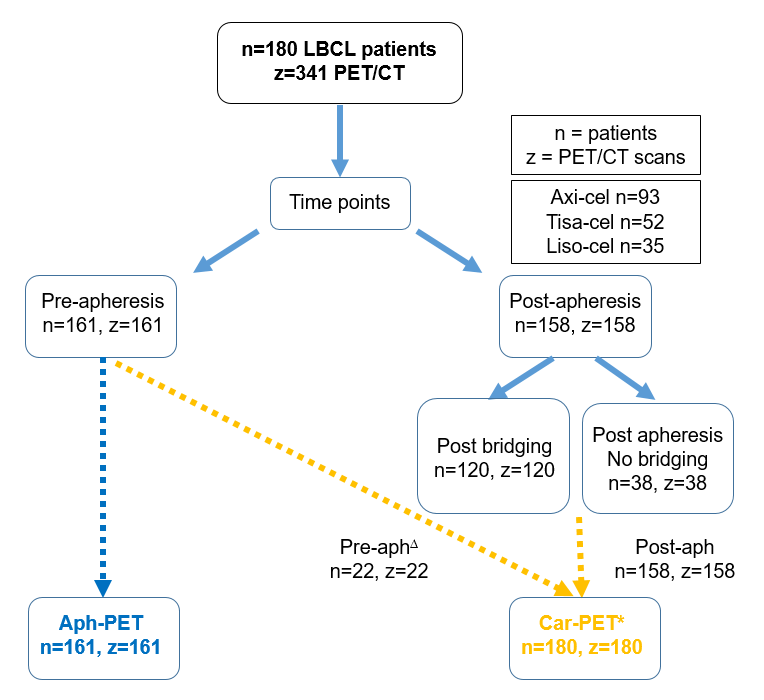


**Supplementary Figure 2. Conventional and novel PET radiomic features.** Original car-[^18^F]FDG PET of a 45-year-old male patient with large B-cell lymphoma. Based on it, conventional features, such as metabolic tumor volume (MTV) and SUVmax (orange dot on MTV, indicated by arrow), as well as novel radiomic features, such as Entropy, Gray Level Non-Uniformity, and Run Length Non-Uniformity, are extracted. Radiomic feature maps illustrate their capturing of different aspects of metabolic tumor heterogeneity. Abbreviation: MTV = metabolic tumor volume.


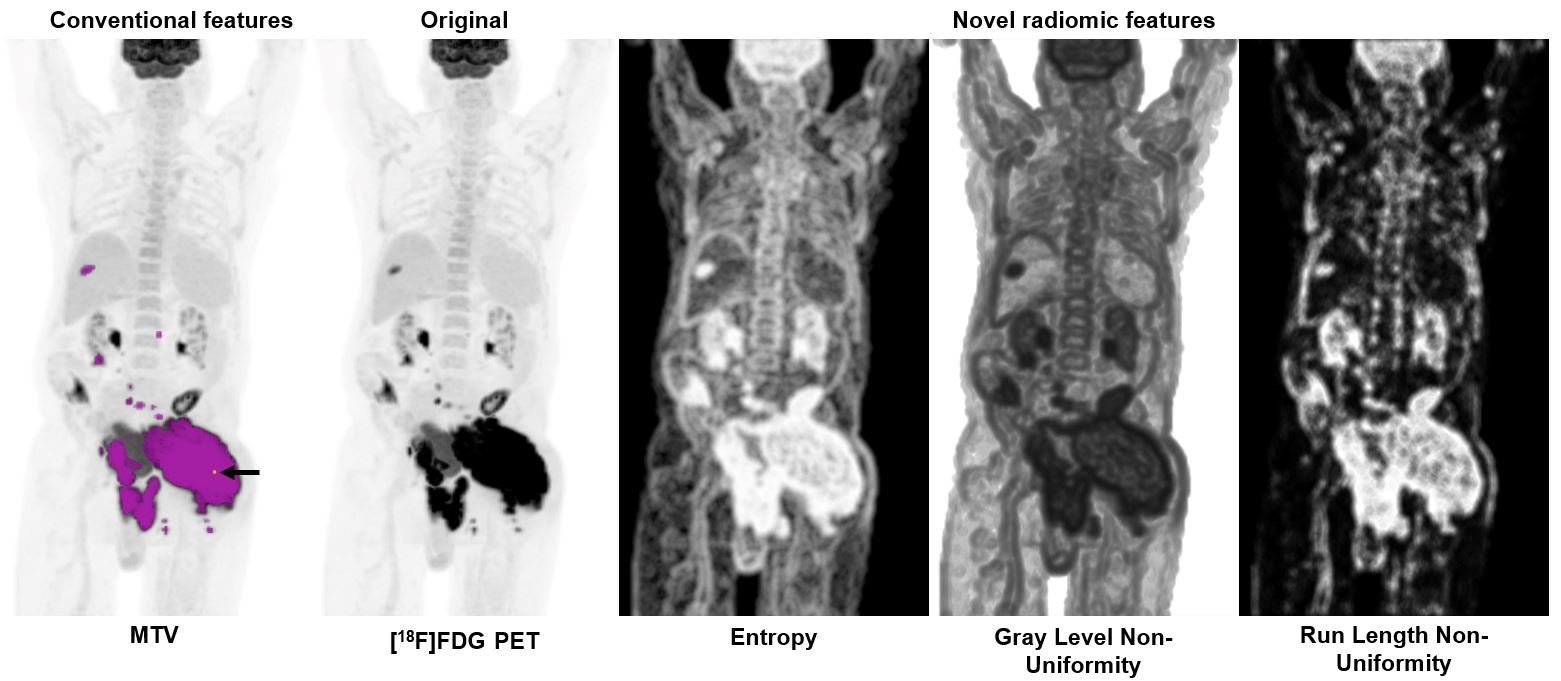


**Supplementary Figure 3.** **Correlations between PET imaging features and markers of inflammation.** Correlation matrix showing color-coded correlation coefficients (Spearman’s rank correlation) for PET imaging features and cytokines for all patients at (**A**) aph-PET and (**B**) car-PET time points. For instance, in the first row, aph-PET MTV moderately correlated with TNF-α (ρ 0.77). Findings indicate that PET features and inflammatory markers are closely related, but complementary to each other. Abbreviations: Aph-PET = pre-leukapheresis PET scan; Car-PET = pre-CAR-T cell infusion PET scan; CRP = C-reactive protein; IL = interleukin; LDH = lactate dehydrogenase; TNF-a = tumor necrosis factor alpha.


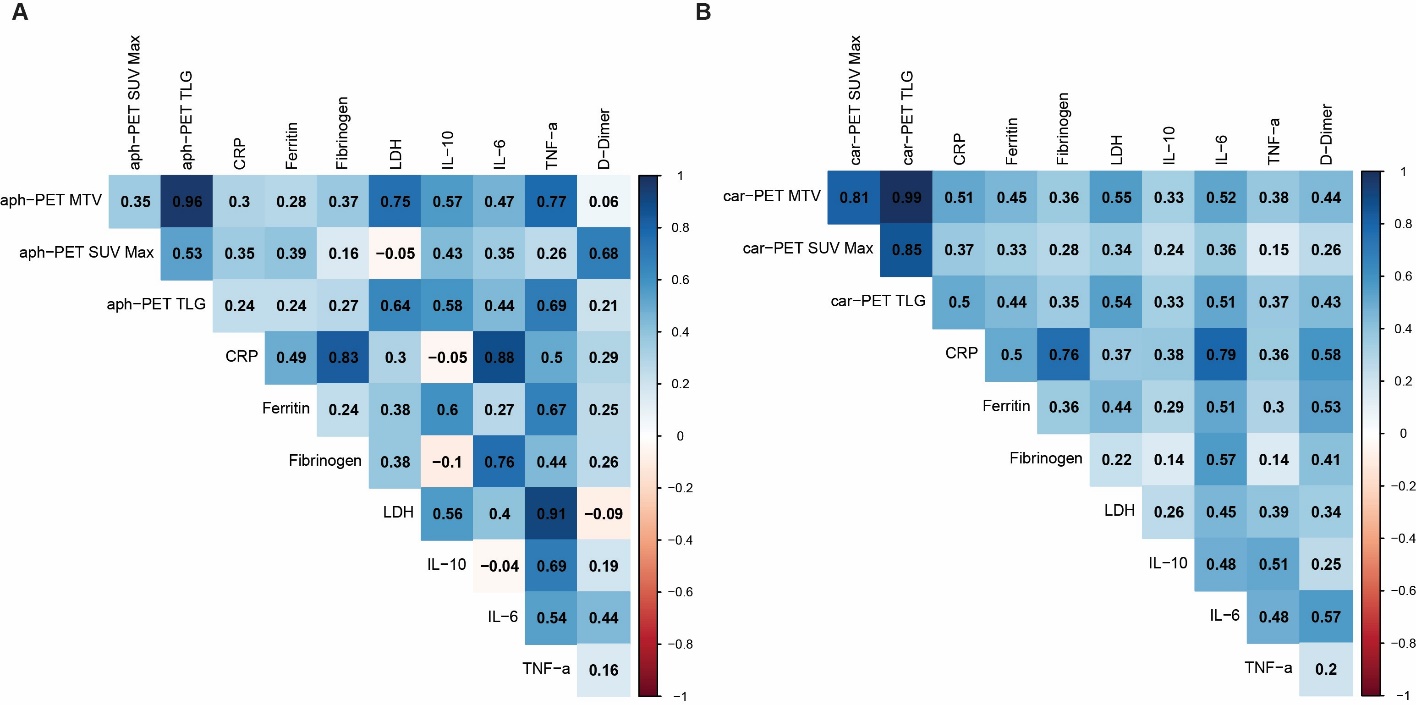

Supplement: Supplementary file 4 — Supplementary Material 4 [file 13045_2024_1540_MOESM4_ESM.docx]
