# Supplementary figures and images for: Conventional and novel [18F]FDG PET/CT features as predictors of CAR-T cell therapy outcome in large B-cell lymphoma
Source: J Hematol Oncol. 2024 Apr 23;17:21. doi: 10.1186/s13045-024-01540-x (PMC11035117; doi:10.1186/s13045-024-01540-x)

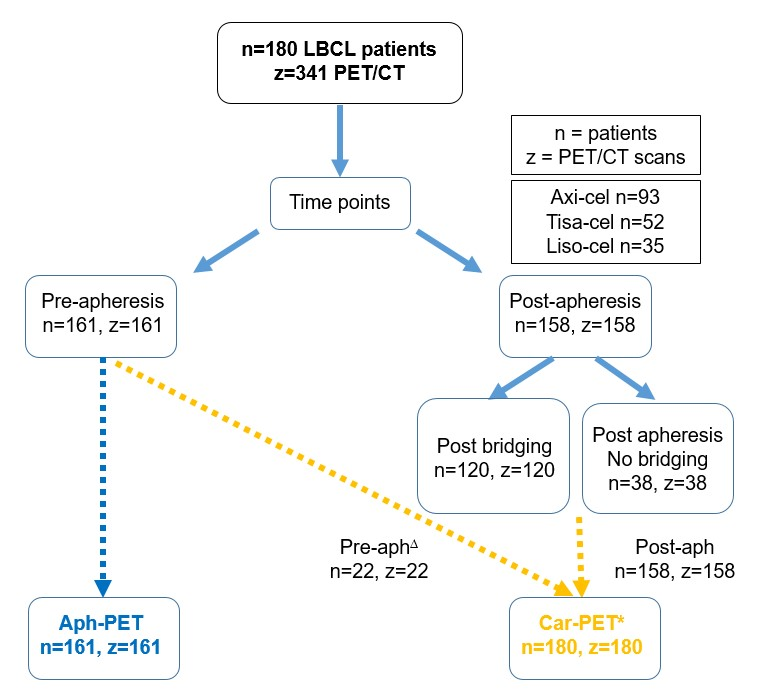

Supplement: Supplementary file 5 — Supplementary Material 5 [file 13045_2024_1540_MOESM5_ESM.tiff]

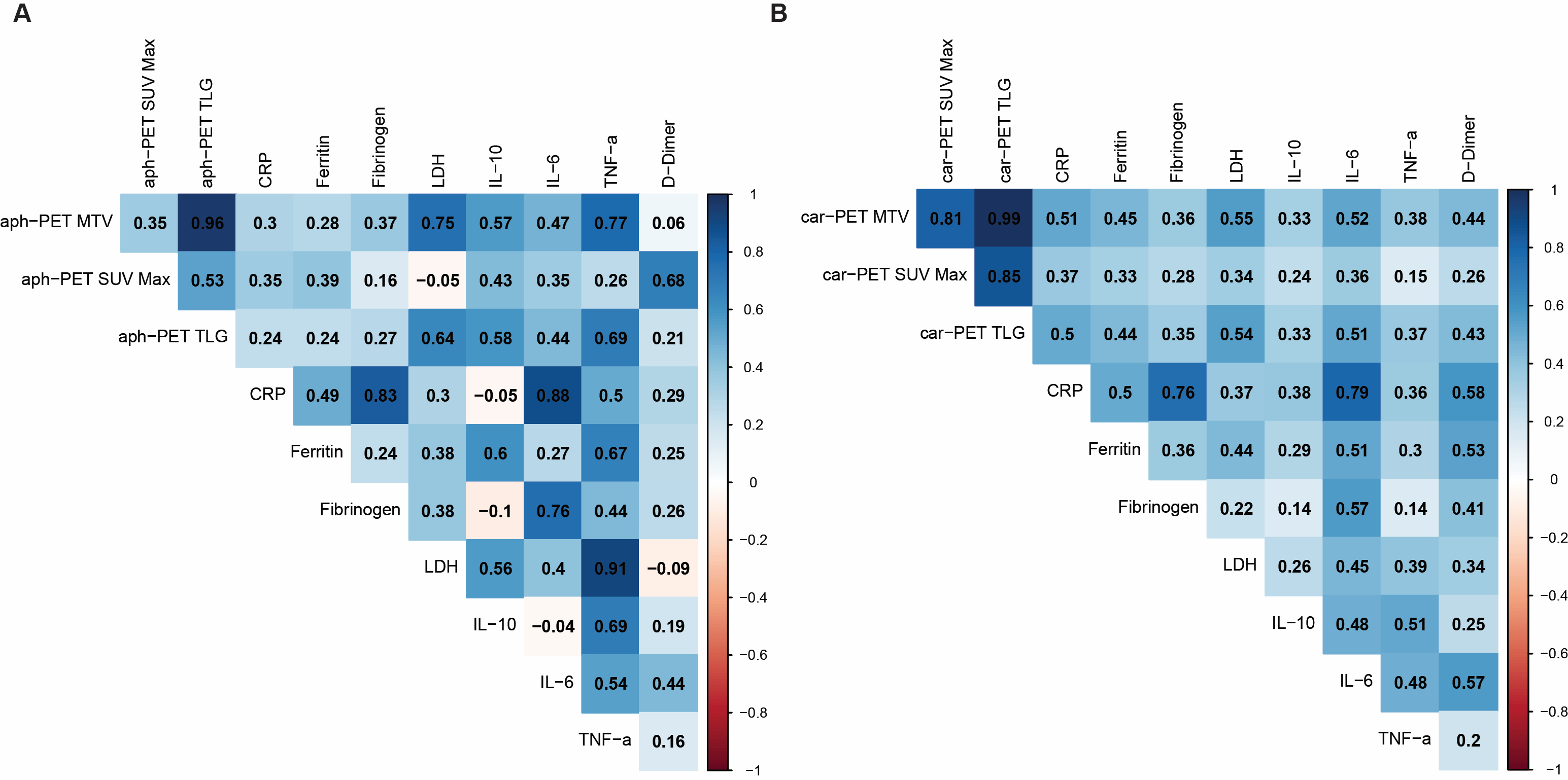

Supplement: Supplementary file 6 — Supplementary Material 6 [file 13045_2024_1540_MOESM6_ESM.tiff]

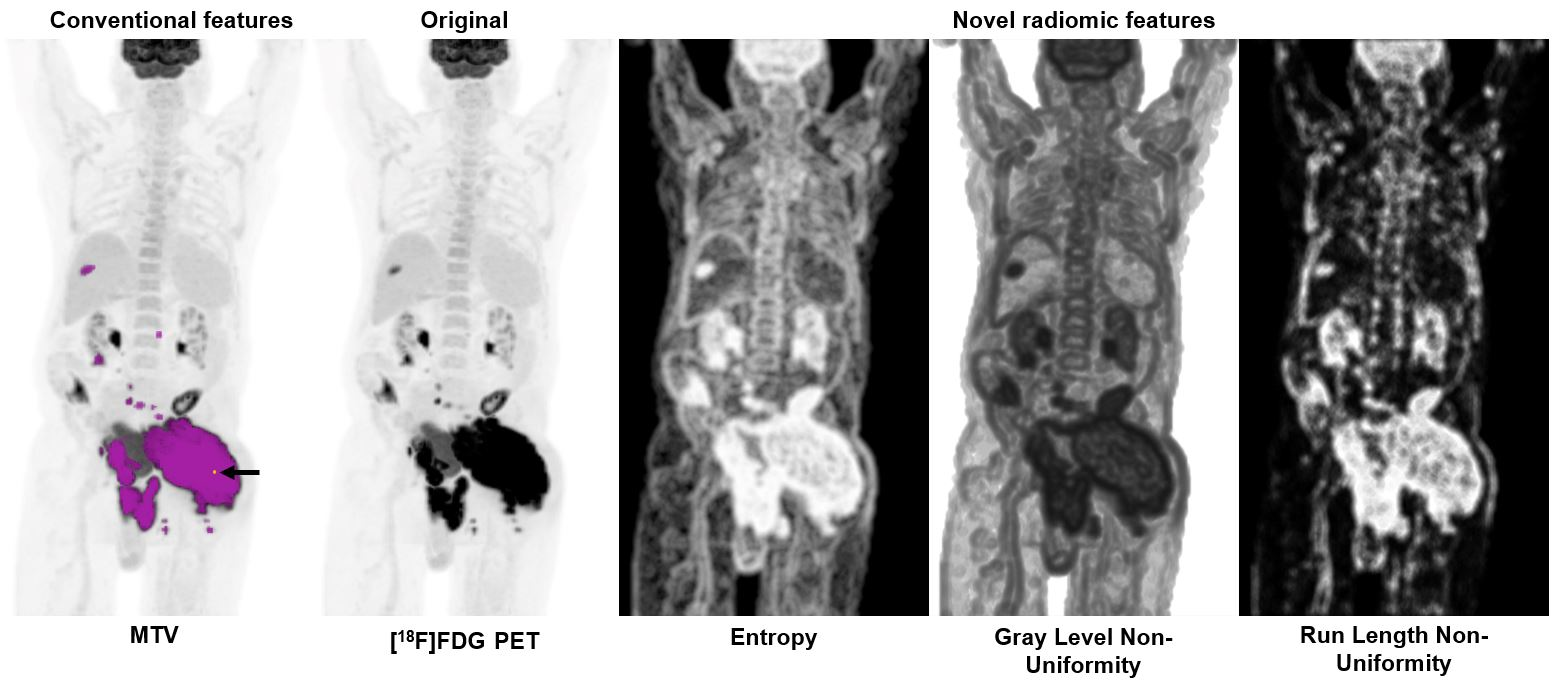

Supplement: Supplementary file 7 — Supplementary Material 7 [file 13045_2024_1540_MOESM7_ESM.tiff]
